# Massive nasal meningoencephalocele in a 4-month-old infant: A case report

**DOI:** 10.1016/j.radcr.2025.03.064

**Published:** 2025-04-17

**Authors:** Shahin Rajaeih, Farshad Riahi, Sam Mirfendereski

**Affiliations:** a ENT and Head and Neck Research Center and Department, The Five Senses Health Institute, Firoozgar Hospital, Iran University of medical Sciences, Tehran, Iran; b Department of Radiology, Isfahan University of Medical Sciences, Isfahan, Iran

**Keywords:** Meningoencephalocele, Encephalocele, Nasal obstruction

## Abstract

A massive nasal meningoencephalocele is a rare congenital anomaly characterized by the herniation of brain tissue and meninges through a defect in the skull base into the nasal cavity. It typically manifests as nasal obstruction and respiratory distress in infancy and is diagnosed via imaging techniques such as magnetic resonance imaging, often necessitating surgical intervention for treatment. This document outlines the endoscopic treatment of a 4-month-old child with a significant nasal meningoencephalocele.

## Introduction

Frontoethmoidal encephaloceles are uncommon congenital malformations characterized by the herniation of brain tissue and meninges via a hole in the skull base [1]. A frontoethmoidal encephalocele is a rare kind, with few known examples globally. These anomalies can lead to various complications in babies, such as nasal obstruction and respiratory distress, and are frequently identified within the first year of life [2]. The prevalence of encephalocele varies between 1 in 3000 and 1 in 5000 live births, but basal cephaloceles are far uncommon, occurring around 1 in 35, 000 live births [3]. The medical management of these situations necessitates meticulous evaluation of the surgical strategy and a multidisciplinary approach to treatment [4].

Here, we describe the endonasal endoscopic management of a 4-month-old infant with a massive nasal meningoencephalocele.

## Case presentation

A 4-month-old infant presented to our clinic with nasal obstruction and difficulty swallowing. Physical examination and laboratory data were normal. Endoscopic examination revealed a nasal mass that occupied the entire left nasal cavity. Magnetic resonance imaging (MRI) was performed to characterize the lesion further.

**Fig. 1:**
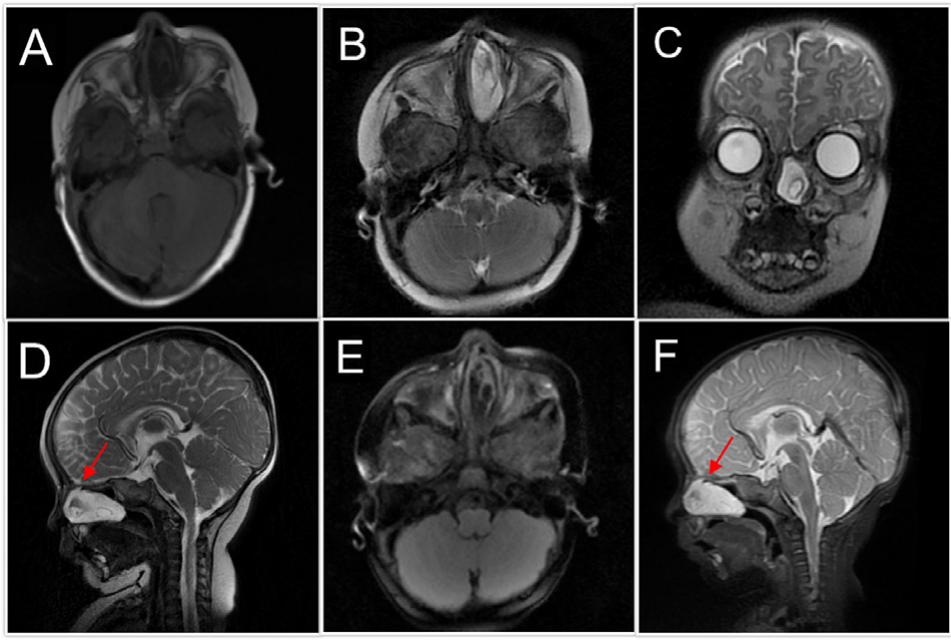
MRI imaging: (A) Axial T1, (B) Axial T2, (C) Coronal T2, (D) Sagittal T2, (E) Axial FLAIR, and (F) Sagittal STIR images show a left nasal cavity lesion with a low signal on T1 and high signals on T2 and STIR with loss of signal on FLAIR. A small hole in front of the skull base (foramen cecum) is indicated by arrows.

**Fig. 2:**
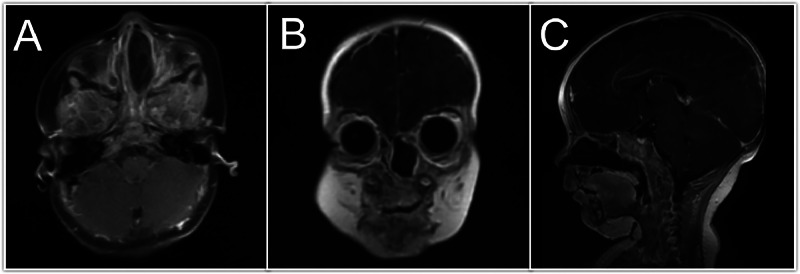
In postcontrast images (A) Axial, (B) Coronal, and (C) Sagittal, no contrast enhancement was observed in the left nasal mass.

On MRI, the mass demonstrated specific signal characteristics. On T2-weighted images, the lesion was homogeneously hyperintense, with signal intensity similar to that of the cerebrospinal fluid (CSF) (Fig. 1). On T1-weighted images, the lesion was hypointense relative to the brain parenchyma (Fig. 1). Importantly, on Fluid-Attenuated Inversion Recovery (FLAIR) images, the lesion showed complete signal suppression, consistent with the CSF content (Fig. 1). The mass was well circumscribed with smooth margins and had a round to ovoid shape. A subtle linear tract of tissue with a similar signal intensity was observed extending from the lesion towards a small defect in the anterior skull base at the foramen cecum. Postcontrast T1-weighted images revealed no enhancement within the lesion or along the tract (Fig. 2). The absence of enhancement was a key finding that helped differentiate the lesion from potentially enhancing inflammatory or neoplastic processes. The presence of a skull base defect at the foramen cecum, coupled with MRI signal characteristics, strongly suggested a diagnosis of left frontoethmoidal meningoencephalocele (Fig. 2).

The patient had endoscopic surgery to remove the nasal mass and reconstruct the anterior cranial base defect with a fat graft inlay and a fascia lata onlay. The pathology report confirmed the diagnosis of meningoencephalocele.

## Discussion

Our case report describes a 4‐month‐old infant who initially presented with nasal obstruction and dysphagia. Clinical and imaging findings eventually led to the diagnosis of basal meningoencephalocele. The patient's symptoms, namely, nasal obstruction and respiratory difficulties, are consistent with the typical presentation of nasal encephaloceles in infants, where such issues frequently manifest during the first year of life. Moreover, the absence of other congenital anomalies, such as hypertelorism, cleft lip/palate, or corpus callosum agenesis, emphasizes the importance of considering meningoencephalocele even when these additional syndromic features are absent [5,6].

MRI is a fundamental component of the diagnostic process. MRI revealed a high-signal lesion within the nasal cavity, a deficit at the foramen cecum, and homogeneous hyperintensity on T2-weighted images, coupled with hypointensity on T1-weighted images. Complete signal suppression on FLAIR and the absence of contrast enhancement further corroborated the interpretation that the lesion contained CSF [7]. It is imperative to recognize even minor bony defects near the foramen cecum, as this region is a known predilection site for skull base anomalies that facilitate herniation of the brain tissue and meninges. Although encephaloceles are typically classified based on the location of the skull base defect—categorized as transethmoidal, spheno-orbital, sphenomaxillary, or transsphenoidal— [1] the lesion in this patient was most accurately characterized as a basal encephalocele, given its proximity to the foramen cecum.

The differentiation of meningoencephaloceles from other pediatric nasal masses, such as nasal polyps, dermoid cysts, and gliomas, is contingent upon distinct MRI signal characteristics and the identification of the specific skull base defect [3]. Even in the absence of overt congenital syndromic features or endocrine disturbances, the presence of a CSF-like signal and subtle tissue tracts connecting to a bony defect strongly suggest the possibility of meningoencephalocele [8]. Although endocrine disorders, including hypopituitarism, deficiencies of antidiuretic hormone or growth hormone, and the requirement for steroid medication, have been documented in association with such encephaloceles, the patient in this study did not exhibit endocrine abnormalities. This observation highlights the notion that basal encephaloceles may manifest subtly until significant herniation occurs, with any potential hypothalamo-pituitary dysfunction more likely to be observed postoperatively than as an initial presenting feature.

Pathological examination following endoscopic resection of the lesion confirmed the diagnosis, corroborating the MRI findings and facilitating appropriate management. Surgical intervention was meticulously planned by a multidisciplinary team comprising pediatric neurosurgeons, otolaryngologists, radiologists, and anesthesiologists. Given the well-defined CSF-like lesion and small bony defect at the foramen cecum, the team elected to employ an endonasal endoscopic approach [9]. This technique, noted for its minimal invasiveness and reduced complication rates compared to traditional transcranial or transpalatal approaches, allows for precise dissection around delicate neurovascular structures, preserves cosmetic outcomes, and decreases overall hospitalization duration [10].

Among the various surgical techniques available for the repair of encephaloceles, including transcranial and transpalatal methods, the endoscopic approach is increasingly favored, particularly for older children and adolescents, owing to its minimally invasive nature and reduced morbidity [9,11]. Historically, the transcranial technique was common; however, it is associated with a higher risk of complications and a mortality rate exceeding 50%, leading to its decline in favor of more advanced techniques, such as endoscopic repair [12]. In our case, the endoscopic method not only reduced surgical risks but also minimized the potential for visible scarring, thereby ensuring an optimal cosmetic outcome.

In conclusion, this case illustrates the typical presentation of basal or frontoethmoidal meningoencephalocele in an infant. The diagnostic process, which integrated MRI evaluation with subsequent pathological confirmation and timely selection of an endoscopic surgical approach, aligns with contemporary best practices described in the current literature. Despite the absence of other congenital anomalies or endocrine disturbances, the early presentation at 4 months of age underscores the importance of prompt imaging and a high index of suspicion for achieving a timely and accurate diagnosis, thereby ensuring effective treatment and improved patient outcomes.

## Patient consent

Written informed consent for the publication of this case report was obtained from the patient.
